# BAFF and APRIL signaling in the B-cell lineage: implications for the pathogenesis of ANCA-associated vasculitis

**DOI:** 10.3389/fimmu.2026.1890512

**Published:** 2026-07-21

**Authors:** Yasuhiro Shimojima, Shuhei Yoshida, Haruki Matsumoto, Yuya Sumichika, Tomoyuki Asano, Shuzo Sato

**Affiliations:** Department of Rheumatology, Fukushima Medical University School of Medicine, Fukushima, Japan

**Keywords:** ANCA-associated vasculitis, APRIL, B cells, BAFF, BAFF-R, TACI

## Abstract

Among the immune mechanisms underlying the pathogenesis of antineutrophil cytoplasmic antibody (ANCA)-associated vasculitis (AAV), B-lineage cells play crucial roles by producing ANCA, presenting antigens to T cells, and secreting proinflammatory cytokines. The upregulated signaling pathways of the B-cell activation factor of tumor necrosis factor family (BAFF) and a proliferation-inducing ligand (APRIL), which are broadly produced by several types of myeloid cells, contribute to the activation and survival of autoreactive B cells, leading to disease onset and relapse. Furthermore, elevated levels of soluble BAFF and APRIL persist even in patients who achieve clinical remission following conventional induction therapies such as cyclophosphamide and rituximab (RTX), resulting in the cause of relapse. Active AAV is characterized by expansions of specific phenotypes in circulating B-cell lineages, including plasmablasts and plasma cells. Conversely, reductions in transitional, memory, and regulatory B cells are observed. Significant expressions of affinity receptors binding to secreting BAFF/APRIL, including decreased BAFF receptor expression on memory B cells and transitional B cells or increased transmembrane activator and calcium-modulator and cyclophilin ligand interactor (TACI) expression on transitional B cells and plasmablasts/plasma cells, are also observed during acute and remission AAV. Enhanced BAFF and APRIL signaling through these receptors, especially TACI, activates the intracellular nuclear factor-κB pathway in B cells, promoting their proliferation and ANCA production. B-cell depletion therapy using RTX is effective; however, it requires repeated administration to maintain remission, which can occasionally lead to infection. Belimumab, a BAFF-inhibiting monoclonal antibody, alone has shown limited efficacy in preventing relapse of AAV. Targeting BAFF/APRIL signaling pathways and their binding receptors, along with the downstream molecular pathways in targeted B cells, is essential for developing novel therapeutic strategies aimed at achieving complete and sustained AAV remission.

## 1 Introduction

Antineutrophil cytoplasmic antibody (ANCA)-associated vasculitis (AAV) is a systemic autoimmune disorder pathologically characterized by a pauci-immune necrotizing vasculitis affecting small- to medium-sized vessels (1, 2). AAV comprises three major types: microscopic polyangiitis (MPA), granulomatosis with polyangiitis (GPA), and eosinophilic granulomatosis with polyangiitis (EGPA), all of which cause severe organ damage and life-threatening complications. The development of inflammatory vascular damage is attributed to the priming of the innate immune system, which leads to immunological crosstalk mediated by ANCA directed against myeloperoxidase (MPO) and proteinase 3 (PR3) (2, 3). These interactions activate neutrophils and the complement pathway, promoting the formation of neutrophil extracellular traps and increasing production of proinflammatory cytokines, chemokines, and reactive oxygen species (ROS). In addition to these mechanisms, various causal inducers such as polygenic genetic susceptibility, epigenetic dysregulation, and environmental factors contribute to AAV development. MPO- and PR3-ANCA are pivotal in AAV pathogenesis and are crucial determinations of clinical classification (4–6).

Autoreactive B cells play a crucial role in AAV through ANCA production and immunopathological pathways. These cells can act as antigen-presenting cells to T cells and produce not only pro-inflammatory cytokines, such as interleukin (IL)-6 and tumor necrosis factor (TNF)-α, but also anti-inflammatory cytokine IL-10 (7–9). Recent studies have demonstrated the potential benefits of addressing the immunopathological aspects of the B-cell lineage in AAV. Moreover, therapeutic agents, such as cyclophosphamide, which target the autoreactive response of B cells, and rituximab (RTX), which depletes these cells, have demonstrated beneficial effects in inducing remission (10–14). The efficacy of maintenance therapy with RTX has also been demonstrated in achieving remission during the long clinical course of AAV (15–19). However, AAV relapse remains common after withdrawal of RTX, and the persistence of CD19^+^ B cells and ANCA positivity is strongly associated with relapse risk (20). These results suggest that residual B cells in lymphoid tissues and bone marrow, including long-lived plasma cells and plasmablasts that lack CD20 expression, may contribute to disease relapse (21). In the context of reconstructive immune response, upregulation of the B-cell activation factor of TNF family (BAFF) and a proliferation-inducing ligand (APRIL) signaling pathway, along with impaired immune tolerance in the B-cell lineage, has emerged as a key mechanism underlying B-cell reconstitution and disease relapse. Both BAFF and APRIL are produced by several types of myeloid cells, including macrophages, neutrophils, monocytes, dendritic cells, and activated acquired immune cells (22–24). Elevated levels of soluble BAFF and APRIL have been associated with disease activity and organ involvement in some studies of AAV (25–29). Experimental studies have further demonstrated that ANCA-activated neutrophils produced BAFF after stimulation with PR3-ANCA immunoglobulin G (IgG) (30). BAFF and PR3-ANCA IgG-stimulated primed neutrophils promoted the BAFF-dependent survival of the centroblast cell line derived from Burkitt’s lymphoma, suggesting that increased BAFF can induce the B-cell lineage, contributing to AAV pathogenesis.

This narrative review highlights the characteristics of BAFF/APRIL signaling and the B-cell lineage and discusses therapeutic strategies targeting autoreactive B cells and BAFF/APRIL signaling in AAV.

## 2 Autoreactive B cells: impacts and perspectives as a therapeutic target in AAV

B cells play multiple roles in the immune system that are involved in the development of autoimmune diseases. These roles include not only the production of disease-specific autoantibodies but also the secretion of inflammatory cytokines and the presentation of autoantigens to T cells, thereby stimulating CD4^+^ and CD8^+^ T cells. Autoreactive B cells mediate autoimmunity by modulating myeloid cell functions (31). In particular, their involvement in disease onset and relapse is closely linked to the production of autoantibodies. Pathogenic autoreactive B cells can be effectively depleted by CD20-targeting monoclonal antibodies (mAb), notably RTX, leading to clinical remission in AAV and other autoimmune diseases, such as systemic lupus erythematosus (SLE), rheumatoid arthritis (RA), and neuromyelitis optica spectrum disorder (NMOSD). However, certain B cell subtypes that are not targeted by RTX can persist and continue to produce antibodies, including anti-SS-A, anti-SS-B, and anti-U1-RNP antibodies, while autoantibody-independent B cells may also persist during disease development (32). These sustained antibody responses are associated with disease flares in patients with SLE (33, 34). Although RTX is widely recommended as an induction therapy for AAV and is effective in reducing relapse risk during maintenance (13, 15–17, 35, 36), the suppression of ANCA seroconversion is not a completely reliable indicator for monitoring remission.

**Figure 1:**
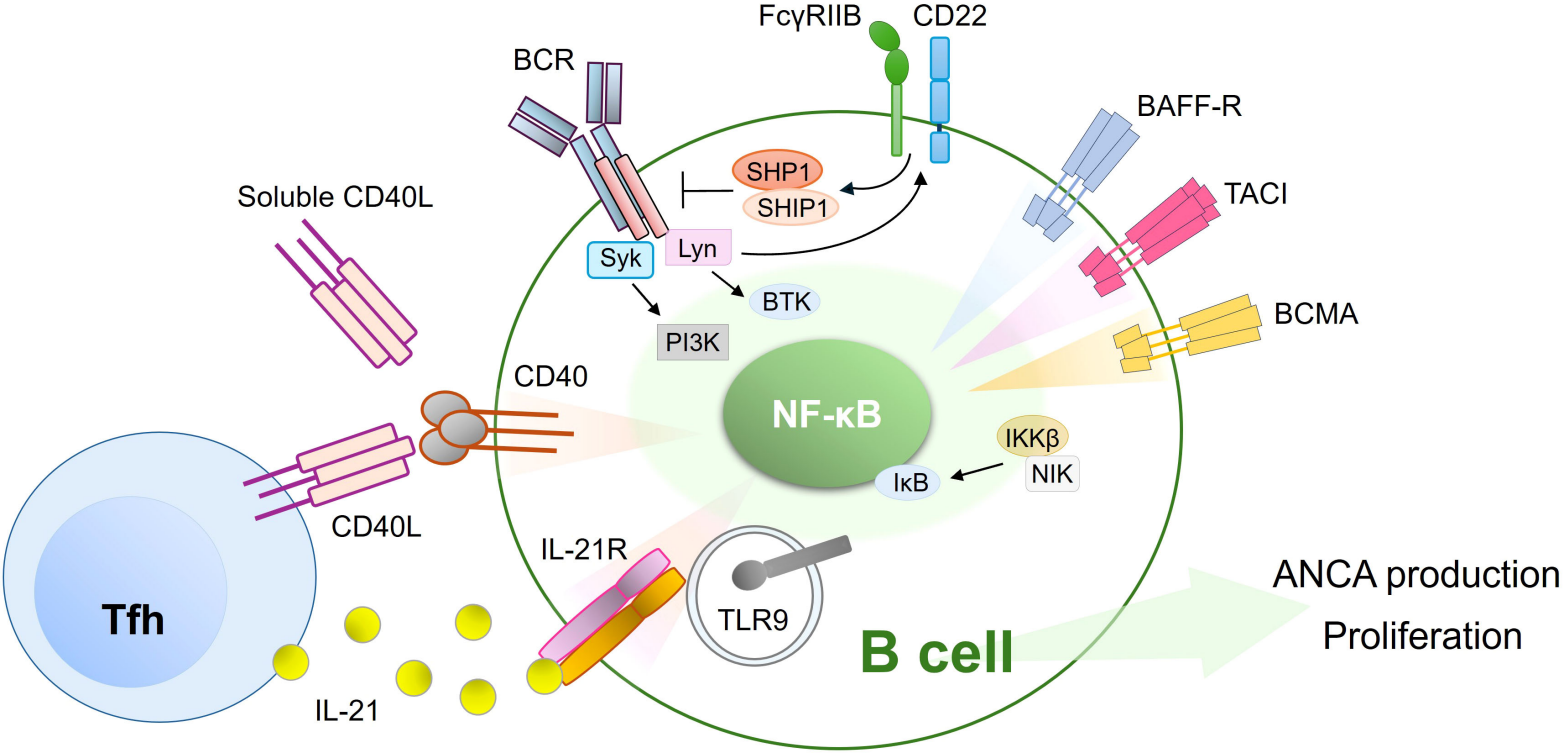
Signaling mediators of B cells in antineutrophil cytoplasmic antibody (ANCA)-associated vasculitis. Follicular helper T (Tfh) cells are associated with B-cell activation by expressing CD40L and producing interleukin (IL)-21, which binds to CD40 and IL-21 receptor (IL-21R) on B cells, respectively. Increased soluble CD40L levels are also observed, which leads to B-cell activation. Upregulation of IL-21R signaling is promoted by toll-like receptor (TLR) 9 stimulation. Signal transduction through the B-cell antigen receptor (BCR), following antigen cross-linking, involves the recruitment and activation of downstream molecules, including Lck/Yes-related novel tyrosine kinase (Lyn), spleen tyrosine kinase (Syk), Bruton’s tyrosine kinase (BTK), and phosphoinositide 3-kinase (PI3K). The Fc fragment of IgG receptor IIb (FcγRIIB) and CD22 are phosphorylated by Lyn, leading to suppression of the BCR signaling by recruiting Src homology region 2 domain-containing phosphatase-1 (SHP1) and SH2 domain-containing inositol 5′-phosphatase 1 (SHIP1). B-cell activation factor of tumor necrosis factor family (BAFF) and a proliferation-inducing ligand (APRIL) signal through their receptors—BAFF receptor (BAFF-R), transmembrane activator and calcium-modulator and cyclophilin ligand interactor (TACI), and B-cell maturation antigen (BCMA)—to activate nuclear factor-κB (NF-κB) in B cells. This process is mediated by NF-κB-inducing kinase (NIK) and inhibitor-of-κB (IκB)-kinase-β (IKKβ), which may affect NF-κB activation by phosphorylating IκB, a negative regulator of NF-κB.

Beyond ANCA production, B cells exert additional effects on AAV pathogenesis. Animal studies suggest that RTX has antibody-independent functions, including B cell apoptosis, an increase in regulatory T cells (Tregs), attenuation of anti-MPO-mediated autoimmunity and kidney damage, and suppression of effector T cell induction in an experimental anti-MPO glomerulonephritis mouse model (37). In patients with active AAV, circulating Tregs are reduced, and their plasticity shifts toward effector phenotypes (38–40). Increased follicular helper T (Tfh) cells, which are physiologically implicated in B-cell maturation and help in their production of high-affinity antibodies (41), and reduced antigen-experienced Tregs recover to circulating levels similar to those in healthy individuals after RTX induction (42). These results suggest that B cells can directly promote T cell responses as antigen-presenting cells in AAV. Clusters of intrarenal B cells have been identified in biopsied kidney tissues from patients with MPO-ANCA nephritis. These clusters act as antigen-presenting cells and form ectopic lymphoid structures, namely tertiary lymphoid organs, including germinal centers (43). Tfh cells promote B cell proliferation and differentiation by expressing CD40 ligand (CD40L), which plays a costimulatory role by binding to CD40 on B cells within germinal centers (**Figure 1**). Increased Tfh cells, producing IL-21, are associated with disease activity and ANCA production (44, 45), and elevated soluble CD40L levels have been observed in patients with AAV compared with those with systemic sclerosis and healthy individuals (46). These findings suggest that Tfh cells, which produce IL-21 and express CD40L, help B cells via the IL-21 receptor (IL-21R) and CD40 stimulation (8, 47). Increased IL-21R expression on memory B cells has been reported in patients with GPA compared to healthy individuals, which is promoted by toll-like receptor (TLR) 9 stimulation and CpG-oligodeoxynucleotide (ODN), suggesting that antigen-experienced B cells become more responsive to IL-21 in GPA (48). TLR9 stimulation by CpG-ODN also influences B cell proliferation, promoting plasma cells and ANCA production (49). The B-cell antigen receptor (BCR) is a transmembrane protein complex on B cells that recognizes specific antigens and transmits signals essential for B-cell survival, maturation, and differentiation into antibody-secreting plasma cells and memory B cells. After cross-linking with antigens, BCR signaling is mediated by downstream molecules, including Lck/Yes-related novel tyrosine kinase (Lyn), spleen tyrosine kinase (Syk), Bruton’s tyrosine kinase (BTK), and phosphoinositide 3-kinase (PI3K) (50). Recent studies have demonstrated reduced expression of the Fc fragment of IgG receptor IIb (FcγRIIB), which is the predominant isoform of FcγR expressed on B-lineage cells, on plasma cells in MPA (51) and on naïve B cells in GPA, but increased expression of FcγRIIB on transitional B cells in GPA (52). In addition, upregulation of CD22 and downregulation of CD21 are observed broadly on various differentiated stages of B cells in patients with GPA, with FcγRIIB and CD22 expression levels associated with disease activity of AAV (52). FcγRIIB and CD22 can be phosphorylated by Lyn, leading to suppression of the BCR signal through the recruitment of Src homology region 2 domain-containing phosphatase-1 (SHP1) and SH2 domain-containing inositol 5′-phosphatase 1 (SHIP1) phosphatases (53, 54). This suggests that dynamic regulation of these molecules contributes to the pathogenesis of AAV. Syk is a pivotal kinase in the early downstream signal of BCR, leading to the activation of nuclear factor-κB (NF-κB) via BTK and PI3K cascade, nuclear factor of activated-T cells (NFAT), and p38 mitogen-activated protein kinases (MAPK) signaling. Significantly increased levels of phosphorylated Syk have been observed in circulating neutrophils and monocytes in patients with active AAV compared with those in AAV remission and healthy individuals (55). The upregulation of phosphorylated Syk in infiltrating immunocompetent cells and inflamed renal tissue has also been demonstrated in patients with AAV and mouse models (55, 56).

Taken together, these results suggest that AAV pathogenesis is closely related to dysregulated autoreactive B cell responses. These processes involve the specific expression of key receptors, certain ligations, relevant signaling molecules, and transcriptional regulators that play roles in cellular activation. These cells are activated by antigen presentation and upstream stimulation, leading to ANCA production. Autoreactive B cells also play a role in antigen presentation, contributing to T-cell activation in the immunopathological pathway of AAV. Although these cells represent key therapeutic targets and can be effectively depleted by RTX, their restoration following treatment likely contributes to disease relapse.

## 3 Phenotypical characteristics of B cells in AAV

### 3.1 Alterations of B-cell subsets

Circulating total B cells are increased or unchanged in patients with active AAV (26, 57–61), but significantly decreased in remission (26, 58, 59, 62), compared with healthy individuals (**Table 1**). Naïve B cell subsets, including CD19^+^CD27^-^CD38^-/+^, CD19^+^CD27^-^CD38^-/dim^, and CD19^+^CD20^-/+^CD27^-^CD38^-/+^ cells, are increased in patients with active and/or remission AAV compared with healthy individuals (52, 60, 62, 63). Another naïve subset of B cells, characterized by CD19^+^IgD^+^CD27^-^ cells, decreases during remission (59) but increases during relapse (21). Immature B cells are reduced in remission compared to healthy individuals (26). Transitional B cells, CD19^+^CD27^-^CD38^high^ cells, are decreased in patients with active AAV but return to levels comparable to those in healthy individuals during remission (52, 60, 63). In patients with GPA in remission, significantly higher frequencies of this subset are associated with subsequent relapse (62). However, there was no significant difference in the frequency of these cells between patients without future relapse and healthy individuals. A decrease in another type of transitional B cells, the CD19^+^IgM^++^CD38^++^ population, was also shown compared to healthy individuals, while the levels increased after RTX treatment (59). The memory B cell subset of CD19^+^CD24^high^CD38^low^ cells decreased in patients with active AAV and increased to levels seen in healthy individuals (64). In the population of CD19^+^ cells, higher frequencies of switched and exhausted memory B cells are observed in patients with AAV after treatment compared with healthy individuals, particularly CD95^+^ exhausted memory B cells (69). During AAV relapse, switched and unswitched memory B cells show significant decreases, specifically CD19^+^IgD^-^CD27^+^ and CD19^+^IgD^+^CD27^+^ populations, respectively (21). Other memory B cell phenotypes, including CD19^+^CD27^+^CD38^-/low^ and CD19^+^CD20^-/+^CC27^+^CD38^-/low^, are lower in patients with active AAV than in healthy individuals (52, 60). These phenotypes remain low even in remission (26, 52, 60, 62, 63). The frequency of memory B cells is inversely correlated with the intracellular expression of IL-10 in the total B cell population (64).

**Table 1:** Characteristics of circulating B-cell phenotypes in ANCA-associated vasculitis.

B-cell subset	Phenotypes	Acute AAV		Rem AAV	References
		vs. healthy	vs. Rem AAV	vs. healthy	
Total B cells	CD19+	ND	high	low	(26)
		ND	high	low	(59)
		ND	ND	ND	(60)
		high	―	―	(61)
		―	―	low	(62)
		―	―	ND	(63)
	CD3-CD19+	ND	high	low	(58)
	CD3-CD56-CD19+	high	―	―	(57)
Naïve B cells	CD19+CD27-CD38-/+	high	ND	high	(60)
		―	―	high	(63)
	CD19+CD27-CD38-/dim	―	―	high	(62)
	CD19+CD20-/+CD27-CD38-/+	high	―	―	(52)
	CD19+IgD+CD27-	high	―	―	(9)
		ND	high	low	(59)
		high	―	―	(61)
Immature B cells	CD19+CD27-CD38+	ND	ND	low	(26)
Transitional B cells	CD19+CD27-CD38high	low	low	ND	(60)
		―	―	low^*1^ or ND^*2^	(62)
		―	―	ND	(63)
	CD19+CD20+/-CD27-CD38high	low	―	―	(52)
	CD19+IgM++CD38++	low	low	high^*3^	(58)
Memory B cells	CD19+CD24highCD38low	low	low	ND	(64)
	CD19+CD27+CD38-/low	low	ND	low	(60)
		―	―	low	(62)
		―	―	low	(63)
	CD19+CD20-/+CC27+CD38-/low	low	low	low	(52)
	CD19+CD27+CD38-	ND	high	low	(26)
	CD19+IgD-CD27+	ND	low	low	(59)
		low	―	―	(9)
Plasmablasts/Plasma cells	CD19+CD27+CD38+	high	high	low	(26)
Plasmablasts	CD19+CD20-/+CD27highCD38high	high	high	―	(52)
	CD19+CD27++CD38++	low	low	low	(59)
	CD19+IgD-CD27highCD38high	high	high	ND	(65)
Plasma cells	CD3-CD56-CD19+CD27highCD38highCD138+	high	―	―	(57)
	CD19+CD20-/+CD27+CD38+	low	―	―	(52)
Regulatory B cells	IL-10+CD19+	low	ND	low	(66)
		low	low	ND	(67)
	CD19+CD24highCD38high	ND^*4^ or low^*5^	high^*4^ or ND^*5^	low	(64)
		low	low	ND	(60)
		ND	ND	ND	(68)
	CD19+CD24highCD27+	low	ND	low	(60)
		―	―	low	(63)
		low	ND	low	(68)
	CD19+CD5+CD24highCD38high	low	low	ND	(67)
	CD19+CD20-/+CD24highCD27+	low	low	low	(52)

AAV, antineutrophil cytoplasmic antibody-associated vasculitis; Rem, remission; Healthy, healthy individuals; ND, not different. ^*1^in patients without relapse, ^*2^in patients having future-relapse, ^*3^in patients treated with RTX, ^*4^in patients with MPO-ANCA, ^*5^in patients with PR3-ANCA.

### 3.2 Plasmablasts and plasma cells in AAV

Circulating plasmablast and plasma cell subsets, characterized by CD19^+^CD27^+^CD38^high^ cells, are increased during remission and are linked to an increased risk of relapse in patients with GPA (62). Additionally, in cases of active nephritis, elevated frequencies of these cells are observed in urine, along with plasma cell infiltration in biopsied renal tissue. In active AAV, specific phenotypes, including CD19^+^CD27^+^CD38^+^, CD19^+^CD20^-/+^CD27^high^CD38^high^, and CD3^-^CD56^-^CD19^+^CD27^high^CD38^high^CD138^+^ cells, are significantly increased compared with healthy individuals (26, 52, 57). Conversely, other phenotypes, including CD19^+^CD27^++^CD38^++^ and CD19^+^CD20^-/+^CD27^+^CD38^+^ cells, are significantly decreased (52, 59). The plasmablast subset, CD19^+^IgD^-^CD27^high^CD38^high^ cells, is significantly increased in active GPA compared to that in remission and healthy individuals and is correlated with the severity of renal involvement (65).

### 3.3 B-cell expression: RTX affects or ANCA involvement

RTX treatment reduces levels of circulating B cells, including CD19^+^ cells, immature B cells, memory B cells, plasmablasts, and plasma cells (26). However, residual memory and plasma cells persist after RTX administration (70), and some phenotypes are correlated with serum ANCA-IgG levels, suggesting persisting ANCA-specific memory B cells. The levels of circulating PR3-positive B cells are significantly higher in PR3-ANCA-positive AAV than in MPO-ANCA-positive AAV and healthy individuals (61, 71). In the circulating PR3-positive autoreactive B cell pool, increased plasmablasts are associated with relapse in PR3-ANCA-positive AAV (21). In contrast, anti-MPO-IgM-expressing B cells, specifically the CD19^+^CD27^+^IgM^+^ memory B-cell subset, are more prevalent in MPO-ANCA-positive AAV compared with healthy individuals (72).

### 3.4 Age-associated B cells in AAV

Age-associated B cells (ABCs) are a heterogeneous subset of B cells implicated in autoimmunity via their production of pathogenic autoantibodies, inflammatory cytokines, and presentation of antigens to T cells, which contribute to the development of autoimmune diseases, such as SLE, RA, multiple sclerosis, and NMOSD (73–76). ABCs, differentiated by BCR along with TLR7/9, interferon (IFN)-γ, and IL-21 stimulations, exhibit high expression of CD11b, CD11c, and T-box transcription factor 21 (T-bet) while displaying low expression or absence of CD21 and CD23 (77, 78). Although, to the best of our knowledge, the impact of ABCs on AAV pathogenesis has not been reported as distinct evidence to date, ABCs may contribute to disease pathogenesis given the central role of B cells in AAV (76).

### 3.5 Regulatory B cells in AAV

Regulatory B cells (Bregs) are essential for maintaining immune tolerance through the production of anti-inflammatory cytokines, particularly IL-10 (79). In both active and remission phases of AAV, IL-10-producing Bregs are decreased (66) (**Table 1**), and their ability to suppress Th1 responses, T-cell production of IFN-γ and TNF-α, is impaired (64, 66). The frequency of Bregs, characterized by CD19^+^CD24^high^CD38^high^ cells, is decreased in acute PR3-ANCA-positive AAV and remission compared with healthy individuals and is positively correlated with IL-10 expression in total B cells (64). However, findings are inconsistent across studies; one study observed an increase in this Breg phenotype compared with that in healthy individuals after RTX induction (64). Others reported a reduction of this Breg phenotype in patients with acute AAV compared with those in remission and healthy individuals (60) and no difference among acute AAV, remission, and healthy individuals (68). Another phenotype of Bregs, characterized by CD19^+^CD24^high^CD27^+^ cells, is decreased in both acute and remission phases of AAV (60, 63, 68). CD5^+^CD24^high^CD38^high^ Bregs in the CD19^+^ population decrease during acute AAV, while those increase during remission and are correlated with reduced serum levels of ANCA (67). IL-10 production from B cells can recover to comparable levels of healthy individuals during AAV remission (60). Bregs in remission GPA can inhibit T-cell proliferation but not IFN-γ and TNF production from T cells (68).

Collectively, active AAV is characterized by increases in naïve B cells, plasmablasts, and plasma cells, alongside decreases in transitional B cells, memory cells, and Bregs. Although findings vary due to different gating strategies or phenotypes used in flow cytometry analysis for evaluating B-cell subset characteristics across studies, these phenotypic alterations likely contribute to AAV immunopathogenesis. Moreover, persistent phenotypic abnormalities in B cells may contribute to disease relapse, even after clinical remission.

## 4 BAFF and APRIL as crucial inducers of B-cell activation in AAV pathogenesis

### 4.1 Expression status throughout the clinical course of AAV

**Figure 2:**
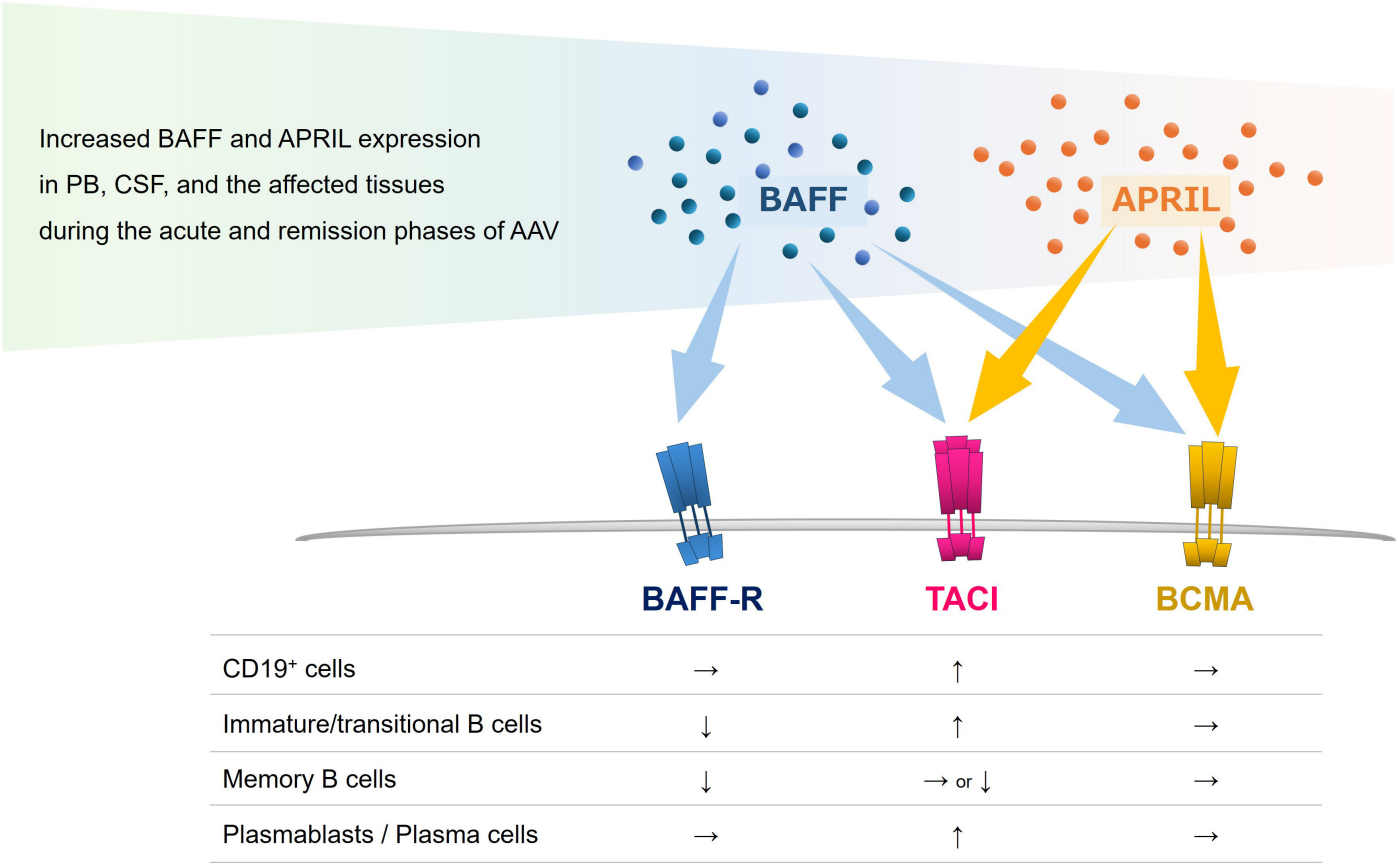
B-cell activation factor of tumor necrosis factor family (BAFF) and a proliferation-inducing ligand (APRIL), and their affinity binding receptors in antineutrophil cytoplasmic antibody (ANCA)-associated vasculitis (AAV). BAFF and APRIL are highly expressed in peripheral blood (PB), cerebrospinal fluid (CSF), and biopsied tissue from involved organs in the acute and remission phases of AAV. BAFF receptor (BAFF-R) and transmembrane activator and calcium-modulator and cyclophilin ligand interactor (TACI) are differentially expressed across B-cell subtypes compared with healthy individuals.

BAFF and APRIL, which belong to the TNF ligand superfamily (BAFF: alternatively known as TNFSF13B, BlyS, CD257; APRIL: alternatively known as TNFSF13, CD256), play a pivotal role in promoting B-cell lineage as the related cytokines capable of being survival and activating factors for B cells, leading to subsequent antibody production through immunoglobulin-class switching (22–24, 80, 81). BAFF also directly stimulates T cells (82), particularly Tfh cells (83), leading to its involvement in B cell activation. Numerous studies have demonstrated increased levels of soluble BAFF and APRIL in the peripheral blood of patients with AAV (26–28, 30, 84–87) (**Figure 2**). Increased BAFF mRNA expression in circulating leukocytes has also been reported in both active and remission AAV compared with healthy individuals (88). Serum BAFF levels do not differ significantly between patients with MPA, GPA, and EGPA (86) or between localized and generalized GPA (87). Elevated BAFF and APRIL levels have also been detected in cerebrospinal fluid (CSF) of patients with ANCA-associated hypertrophic pachymeningitis (28, 29, 89). BAFF and APRIL levels in CSF do not correlate with serum levels; however, they are correlated with the IgG-index, suggesting that BAFF and APRIL may be produced in the central nervous system and may be linked to local B-cell activation in AAV (90). BAFF and APRIL expression has been observed in infiltrating immunocompetent cells, along with activated B cells and PR3-expressing cells, within mucosal tissues of patients with GPA (91). Serum BAFF and APRIL levels remain elevated even during AAV remission, including after B-cell depletion treatment with RTX (25, 26, 30, 59). Moreover, patients treated with RTX exhibit higher serum BAFF levels compared with those without RTX (26).

### 4.2 Production mechanism and relationship with disease activity

Persistent high serum levels of BAFF and APRIL after immunosuppressive therapies have also been shown in other autoimmune diseases (92, 93). These findings suggest that upregulation of BAFF and APRIL may promote circulating B cell repopulation (29), potentially driven by residual pathological B cells in tissues (25). Accordingly, serum levels of BAFF and APRIL may be useful indicators for monitoring AAV remission. Moreover, persistent increases in serum BAFF and APRIL levels, caused by the activation of their producing immunocompetent cells, may lead to a relapse of AAV. Although the mechanisms of BAFF and APRIL production from immunocompetent cells are not fully understood, both in the context of autoimmune diseases and in normal physiological environments, the presence of an NF-κB binding site in the promoter region of BAFF suggests a role of NF-κB in regulating BAFF expression (94, 95). In patients with active AAV, BAFF production by circulating monocytes correlates with NF-κB expression and disease activity (96), as evaluated by the Birmingham vasculitis activity score (BVAS) (97), while APRIL production appears to be influenced by increased intracellular ROS (96). Some studies report correlations between BVAS and serum BAFF or APRIL levels (27, 98), while others show no significant correlations (25, 26, 87). ANCA levels generally do not correlate with serum BAFF or APRIL levels (27, 84, 86), although an inverse correlation between serum BAFF and ANCA levels has been reported in GPA (87). Additionally, a positive correlation between increased serum APRIL levels and memory B cells has been observed in AAV (26).

Taken together, persistent elevation of soluble BAFF and APRIL levels, even during clinical remission, suggests that ongoing BAFF/APRIL signaling abnormalities may contribute to AAV relapse. However, the expression levels of BAFF and APRIL may not always serve as reliable biomarkers for influencing disease activity, as they do not consistently correlate with BVAS.

## 5 BAFF and APRIL signaling through affinity binding receptors in B cells of AAV

### 5.1 Three types of affinity binding receptors

BAFF and APRIL exert their biological effects on B cells by binding to three different types of affinity BAFF/APRIL receptors, which are classified as membrane proteins of the TNF receptor superfamily, including BAFF receptor (BAFF-R; CD268; BR3; TNFRSF13C), transmembrane activator and calcium modulator and cyclophilin ligand interactor (TACI; CD267; TNFRSF13B), and B-cell maturation antigen (BCMA; CD269; TNFRSF17) (7, 81, 99–101). These receptors are expressed on the membrane surfaces of B cells and other types of immunocompetent cells. BAFF has a physiological affinity for BAFF-R, TACI, and BCMA, whereas APRIL ordinarily binds to TACI and BCMA and has no affinity for BAFF-R (7, 81, 100–102). The expression of these receptors varies across B-cell developmental stages. BAFF-R is expressed on B-cell subsets, except for plasma cells and centroblasts in the dark zone of germinal centers, particularly during immature to transitional B cells development (103, 104). TACI is typically expressed on activated B cells, including marginal zone B cells, memory B cells, and plasma cells (103–105). BCMA is also upregulated in these activated B cells and is constitutively expressed on long-lived plasma cells (106–108). Through these affinity binding receptors, BAFF and APRIL promote B-cell survival, activation, and differentiation, thereby contributing to AAV pathogenesis and ANCA production.

### 5.2 Characteristics of BAFF-R, TACI, and BCMA expression

Altered receptor expression has been reported in AAV. Despite the physiological expression of BAFF-R in various B-cell subsets from an early differentiation stage, contributing to their maturation (103, 109, 110), patients with GPA exhibit significantly lower BAFF-R expression on B cells than healthy individuals (48, 52), and decreased expression has been observed across multiple B-cell subsets, including memory, transitional, naïve, and marginal zone (MZ)-like B cells, in both active and remission phases (26, 59). BAFF-R expression is further reduced in transitional, naïve, and MZ-like B cells after RTX maintenance therapy (59). Soluble BAFF levels are inversely correlated with BAFF-R expression in B cells of patients with GPA (48) and in non-plasmablast B cells of patients with treatment-naïve AAV (59). Even in patients with AAV in remission, serum BAFF levels are inversely correlated with BAFF-R expression in CD19^+^ cells, memory B cells, and plasmablasts/plasma cells (26). Positive correlations have been shown between BAFF-R expression in non-plasmablast B cells and circulating total B cells (59), or between circulating memory B cells and their BAFF-R expression (26), in patients with treatment-naïve AAV. The frequencies of circulating CD19^+^ cells, immature B cells, and plasmablasts/plasma cells are positively correlated with BAFF-R expression in patients with AAV in remission (26).

TACI-expressing naïve B cells are increased, while TACI-expressing memory B cells decreased in patients with GPA compared to healthy individuals (48). Another study has reported increased TACI expression in CD19^+^ cells, immature B cells, plasmablasts, and plasma cells in both acute and remission AAV compared with healthy individuals (26). Higher TACI expression in CD19^+^ cells has been observed in patients with ear, nose, and throat (ENT) involvement compared with those without ENT. In contrast, TACI expression decreases in MZ-like and memory B cells during maintenance RTX therapy, although the expression did not differ significantly between patients with treatment-naïve AAV and healthy individuals (59).

BCMA, which has high affinity for APRIL (7, 101), is associated with long-lived plasma cells and persistent antibody production (106, 108, 111). BCMA- and TACI-expressing CD138^+^ plasma cells, along with APRIL expression, have been identified within inflamed nasal tissues of patients with GPA (112). Although no consistent differences in BCMA expression have been reported among the subsets of circulating B cells between patients with AAV and healthy individuals, BCMA expression in memory B cells has been associated with renal function or ENT and decreases during remission (26). Therapeutically, dual targeting of CD19 and BCMA using chimeric antigen receptor (CAR)-T cells has shown promise in other autoimmune diseases, such as SLE, by targeting B-cell subsets that are not eliminated by RTX (113). These findings suggest a potential role for BCMA in refractory disease.

### 5.3 Intracellular signaling pathways via BAFF-R, TACI, and BCMA

BAFF-R, TACI, and BCMA are capable of binding to TNF receptor-associated factor (TRAF) proteins, leading to the activation of NF-κB and the linked p38 MAPK pathway (80). BAFF/APRIL stimulation through BAFF-R, TACI, and BCMA binding to TRAFs activates classical signaling pathways, including NF-κB, activator-protein-1 (AP-1), nuclear factor of activated T-cells (NFAT), and p38 MAPK (80, 114, 115). Alternatively, BAFF-BAFF-R can activate NF-κB through noncanonical signaling (80). Although these signaling pathways have been well characterized experimentally, their precise roles in AAV remain incompletely understood. RNA sequencing of circulating memory B cells from patients with active GPA has demonstrated upregulation of genes involved in both canonical and noncanonical NF-κB signaling compared with patients in remission and healthy individuals (116). Pharmacological inhibition of inhibitor-of-κB kinase β (IKKβ) and NF-κB–inducing kinase (NIK), which regulate canonical and noncanonical NF-κB pathways, respectively, suppresses NF-κB activation and reduces autoreactive B-cell proliferation and PR3-IgG production in response to BAFF, IL-21, and CpG-ODN stimulation (116, 117).

Overall, upregulation of BAFF/APRIL signaling is pivotally linked to the pathogenesis of AAV by promoting autoreactive B cell activation. Altered BAFF-R and TACI expression across B-cell subsets is observed in both acute and remission AAV. The characteristics of BAFF-R, TACI, and BCMA expression vary among different B-cell subsets, and their differences from healthy individuals may be associated with disease pathogenesis. However, their expression statuses do not consistently persist through the overall clinical course. Changes in their expression have been observed based on treatment status, particularly RTX administration. Accordingly, defining the variety of BAFF/APRIL affinity receptors across different clinical statuses may be crucial for identifying therapeutic targets for AAV.

## 6 Treatments targeting autoreactive B cells and BAFF/APRIL signaling

Targeting B cell activation and persistently upregulated BAFF/APRIL signaling, which drive ANCA production and downstream immunopathological interactions, is a fundamental therapeutic strategy for AAV. RTX is a pivotal and reliable agent that depletes pathogenic CD20-expressing B cells to induce and maintain remission. RTX has not demonstrated clear superiority over cyclophosphamide for induction over short-term treatment (12, 13, 15), while RTX has shown superior efficacy compared to cyclophosphamide in patients experiencing a relapse of AAV (13); therefore, its use is supported by robust evidence and clinical guidelines. Meanwhile, cyclophosphamide use is limited by cumulative toxicity, including malignancy risk and reproductive impairment, as well as limited data supporting its use following RTX (11, 118, 119). Clinical trials, including MAINRITSAN1 and RITAZAREM, have demonstrated that RTX is significantly more effective than azathioprine in reducing severe relapse during remission maintenance in MPA and GPA (17, 19), and scheduled RTX administration further reduces relapse risk (18, 120). Accordingly, the 2021 American College of Rheumatology guideline and the 2022 European Alliance of Associations for Rheumatology recommend RTX for maintenance therapy following remission induction with either cyclophosphamide or RTX in strategies, with azathioprine or methotrexate considered as alternatives (10, 11). However, long-lived plasma cells, which do not express CD20, may continue to produce ANCA despite RTX-induced remission (8). As an alternative CD20 depleting agent, obinutuzumab, a glycoengineered type II monoclonal antibody with enhanced antibody-dependent cellular cytotoxicity despite lower complement-dependent cytotoxicity than RTX (121), has shown improved clinical response by inducing B-cell depletion in lupus nephritis (122). A randomized, phase II, double-blind controlled study of obinutuzumab (ObiVas), in which CD19^+^ B cell depletion in nasal tissue, is evaluating its safety and efficacy compared to RTX in PR3-ANCA-positive AAV (123). Early reports suggest potential benefits of obinutuzumab in RTX-intolerant patients and those with refractory disease, with favorable remission rates and acceptable safety profiles (124–126). Considering the role of plasma cells in persistent ANCA production, targeting these cells represents an additional therapeutic approach. Bortezomib, a proteasome inhibitor that is usually used for the treatment of plasma cell dyscrasia, such as multiple myeloma, has shown efficacy for refractory glomerulonephritis in a patient with MPA (127) and a mouse model of MPO-IgG-mediated necrotizing crescentic glomerulonephritis (128). However, obinutuzumab and bortezomib have insufficient evidence as established therapeutic strategies in AAV from well-designed clinical trials. CAR-T cell therapy targeting CD19 (anti-CD19 CAR-T) has emerged as a novel therapeutic strategy for refractory SLE by depleting disease-specific antibody-producing B cells (129). In the MPO-AAV mouse model, anti-CD19 CAR-T cells significantly depleted B cells and plasmablasts, resulting in reduced MPO-ANCA expression and reduced renal injury, and also protected against MPO-ANCA-induced crescentic glomerulonephritis (130). These results suggest the potential of anti-CD19 CAR-T cells in refractory AAV. Favorable effects of anti-CD19 CAR-T cells have been reported in three patients with AAV who were refractory to standard treatments, including cyclophosphamide, RTX, and other immunosuppressants (131–133). Meanwhile, further elucidation of specific B-cell lineages, including their phenotypic and molecular expression related to disease development, may be essential for designing reliably effective CAR-T cells. Anti-CD19 CAR-T cells combined with BCMA-targeting, which is more broadly implicated in depleting B-lineage cells, have been established for the management of SLE (113). CD19 is a pan-B-cell marker that covers all types of B cells; however, its expression can decrease or disappear in mature plasma cells. BCMA targets all antibody-secreting cells, including peripheral short-lived and tissue-resident long-lived plasma cells (111). Moreover, long-lived plasma cells in the bone marrow secrete autoantibodies associated with SLE in animal models (134). In contrast, a recent study using a BCMA-deficient mouse model has demonstrated no significant reduction in plasma cells, suggesting that BCMA may be dispensable for maintaining long-lived plasma cells (107).

Targeting BAFF/APRIL signaling has also been explored. Belimumab, a human IgG1λ monoclonal antibody against soluble BAFF, is approved for SLE and has demonstrated clinical efficacy and safety (135–137). In the clinical study of patients with MPA and GPA (BREVAS trial), belimumab did not significantly reduce relapse rates in AAV (138). The BREVAS trial examined the efficacy of belimumab in combination with azathioprine or low-dose glucocorticoids after remission induction therapy using either RTX or cyclophosphamide for maintaining remission. No relapses were observed in patients receiving belimumab following RTX induction, whereas relapses occurred in those receiving belimumab after cyclophosphamide induction. This suggests that the depletion of CD20^+^ B cells during induction therapy may be crucial for enhancing the remission maintenance efficacy of belimumab, which targets both CD20^+^ and CD20^-^ B cells (139) and neutralizes increased BAFF expression. Accordingly, an ongoing study, COMBIVAS, is evaluating the clinical efficacy of sequential belimumab administration combined with a single cycle of RTX in patients with PR3-ANCA-positive AAV (140). However, APRIL is also implicated in the pathogenesis of AAV and shows a higher affinity for the TACI/BCMA signaling pathway, leading to the development of mature B cells and plasma cells compared to BAFF. Telitacicept, a fusion protein of the IgG Fc fragment and the extracellular domain of TACI, is expected to be a useful agent for achieving remission of SLE, IgA nephropathy, and primary Sjögren’s disease (141–143). A case report described the successful treatment of a patient with GPA with several visceral impairments, including rapidly progressive glomerulonephritis, pulmonary, and ENT lesions, following the sequential administration of telitacicept along with intermittent cyclophosphamide induction (144). However, it is still necessary to rigorously evaluate long-term efficacy and safety through randomized clinical studies to establish it as a proven therapeutic agent. Several ongoing clinical studies on telitacicept, including prospective, open-label, randomized, and controlled trials (TTCAAVREM: NCT05962840; TTCAZAREM: NCT05965284) and one prospective, single-arm, randomized, and open-label exploratory clinical study (TEST-T-AAGN: NCT06656962), are evaluating its efficacy in AAV.

## 7 Conclusions and perspectives

Immature B cells are primarily autoreactive, meaning that they strongly respond to self-antigens. To ensure proper physiological functions, autoreactive B cells are progressively eliminated through central and peripheral tolerance checkpoints. In the central tolerance system, self-reactive B cells can be removed through apoptosis, anergy, or receptor editing, allowing immature B cells in the bone marrow to successfully mature into circulating transitional B cells. In peripheral tolerance, transitional B cells develop into naïve B cells, which ensure self-tolerance and are prepared to respond to foreign antigens. They then migrate towards further mature stages, including memory and antibody-secreting B cells (145–147). Bregs also play a crucial role in peripheral tolerance by restoring immune homeostasis through the regulation of immune responses and suppression of excessive inflammatory reactions. However, this review highlights that these tolerance mechanisms of the B-cell lineage are impaired in AAV. Significantly altered distributions of circulating B-cell subsets, including disease-relevant autoreactive B cells capable of secreting MPO- or PR3-ANCA, are associated with AAV development (61, 71, 72). In addition, increased BAFF/APRIL secretion and altered expression of their receptors, especially BAFF-R and TACI, distinguish patients with AAV from healthy individuals and contribute to AAV pathogenesis. Moreover, persistent upregulation of BAFF/APRIL signaling, along with abnormal receptor expression, has been implicated in AAV relapse. Elevated serum BAFF levels may reflect disrupted feedback regulation following B-cell depletion therapy. This may occur when B-lineage cells expressing BAFF/APRIL-binding receptors are eliminated following RTX treatment, resulting in a lack of affinity binding receptors (25, 148). Accordingly, restoring the physiological status of B-cell communication and regulating BAFF/APRIL signaling are ideal therapeutic goals for complete AAV remission. Although current advanced therapeutic strategies, including B-cell depletion, BAFF/APRIL inhibition, and emerging approaches such as anti-CD19 CAR-T cells, have improved disease control, further characterization of disease-specific B-cell phenotypes and their molecular signaling networks is needed. Such insights will be critical for developing more precise therapies, including optimized CAR-T cell strategies and targeted modulation of the BAFF/APRIL axis.
